# Thermal imaging as a diagnostic tool for superficial venous insufficiency – a systematic review

**DOI:** 10.1177/02683555241301194

**Published:** 2024-11-12

**Authors:** Marwah Salih, Sarah Salih, Benedict R H Turner, Sarah Onida, Alun H Davies

**Affiliations:** Section of Vascular Surgery, Department of Surgery and Cancer, 4615 Imperial College London, London, UK

**Keywords:** Chronic venous disease, vascular surgery, venous disease, duplex ultrasound

## Abstract

**Objectives:**

Evaluation of the literature assessing the use of thermography, a non-invasive imaging modality that detects pathological temperature variation, in recognising superficial venous insufficiency (SVI).

**Methods:**

A systematic review was conducted according to Preferred Reporting Items for Systematic Reviews and Meta-Analyses (PRISMA) guidelines. Articles were screened by two individuals and data was subsequently extracted. Methodological quality was assessed with Cochrane’s risk of bias tool.

**Results:**

Four studies comprising 363 patients were included. Three studies identified increased temperature uptake in veins with incompetent valves on venous duplex (*p* < .05). One study reported sensitivity and specificity of thermal imaging in SVI as 98.30% (95% CI, 95.2%–99.4%) and 100% (95% CI 85.7%–100%) respectively.

**Conclusion:**

Thermal imaging could act as a screening tool in SVI. This review highlights a lack of high-quality prospective studies evaluating the role of thermal imaging as a diagnostic tool that could expedite the assessment of patients.

## Introduction

Chronic venous disease (CVD) is a multifactorial condition that is becoming an increasing burden for patients and healthcare systems.^1–3^ Chronic venous disease in itself can be secondary to reflux within the superficial venous system.^4^ CVD can present with a wide range of symptoms, some of which are non-specific and can therefore lead to delayed diagnosis.^1^ Initial symptoms include aching, leg heaviness, oedema and itchiness, and disease progression may lead to skin changes and ulceration. Symptoms are associated with significant impairment of health-related quality of life at all stages of the disease. Currently, the gold standard investigation for superficial venous insufficiency (SVI) is duplex ultrasound (DUS), recommended for patients after referral to vascular services and review by a vascular surgeon in the UK. DUS assesses the distribution and extent of venous reflux, to allow for subsequent treatment planning.^5^ DUS, however, requires specialised training and can be operator dependent. For these reasons, obtaining DUS is time intensive and delays the time to formal diagnosis and definitive treatment for patients with SVI. The non-specificity of some clinical manifestations of venous disease can also make differentiation from other pathologies difficult and lead to excess DUS requests. The Edinburgh Vein Study observed 13% of female patients reporting leg heaviness and 28.4% reporting leg aching having no evidence of superficial truncal venous insufficiency.^6^

Thermography detects, records, and produces an image (thermogram) of skin surface temperature.^7^ While thermography has existed for decades, advancements in the technology have allowed for a resurgence in investigation of this imaging modality in clinical practice, that has demonstrated promising results.^8^ Thermography is quick, non-invasive and can detect temperature differences and quantify changes in skin temperature, which occur secondary to pathological changes such as inflammation, venous hypertension and increased cellular metabolism. These strengths suggest that thermography could be advantageous, when compared to duplex ultrasound, due to the ease and speed of taking and processing images, without requiring extensively trained personnel. Patients with non-specific symptoms, including heavy and painful legs or legs with rashes/ulcers may have a wide differential diagnoses and would not necessarily need DUS.^9^

Thermography could therefore be used to help streamline and optimise the diagnostic and treatment pathway for patients, improving both the care received and their overall outcomes. Given delays in the referral pathway and the wait between referral to being seen by specialists within the NHS, assessing thermal imaging as a valid point-of-care (POC) test could be key to introducing this test into practice. This systematic review aims to evaluate the current literature describing the use of thermography as a screening tool in SVI.

## Methods

The review was performed in accordance with the Preferred Reporting Items for Systematic Reviews and Meta-Analyses (PRISMA) and registered on PROSPERO prospectively (CRD42023494238). A systematic search was created and performed to identify all relevant articles on the PubMed, EMBASE, Medline and the Cochrane library databases.

### Inclusion and exclusion criteria

Articles reporting on adult patients over 18 years of age with thermal imaging of the lower limbs to assess superficial venous insufficiency were included. Articles that did not include a venous duplex as the comparator for diagnostic imaging were excluded. Case reports, letters and opinion pieces were also excluded. Studies were restricted to English language and human participants only.

### Data extraction and critical appraisal

All studies identified from the initial search were exported into the reference management software programme, Covidence (Veritas Health Innovation, Melbourne, Australia), with all duplicates removed. Two independent reviewers initially screened titles and abstracts, followed by the full texts, against the eligibility criteria (MS, SS). Any disagreements were resolved by a third reviewer (BT). Extracted data included paper and patient demographics, thermal imaging methodology, thermogram temperature findings, diagnostic accuracy, duplex findings and reflux pattern. If sufficient data was found and able to undergo meta-analysis, a random-effects model would have been utilised and heterogeneity would be calculated with the I^2^ statistic.

### Risk of bias

Studies were assessed for methodological robustness using the Cochrane risk of bias (RoB) tool. For non-randomised studies, we assessed bias using the Cochrane risk of bias in non-randomised studies of interventions (ROBINS-I) tool under the following bias domains: confounding, selection, intervention classification, missing data, outcome measurement and selecting reporting. Discrepancies were discussed by the two reviewers until consensus was reached, with any disagreements were resolved by a third reviewer.

## Results

**Figure 1.**
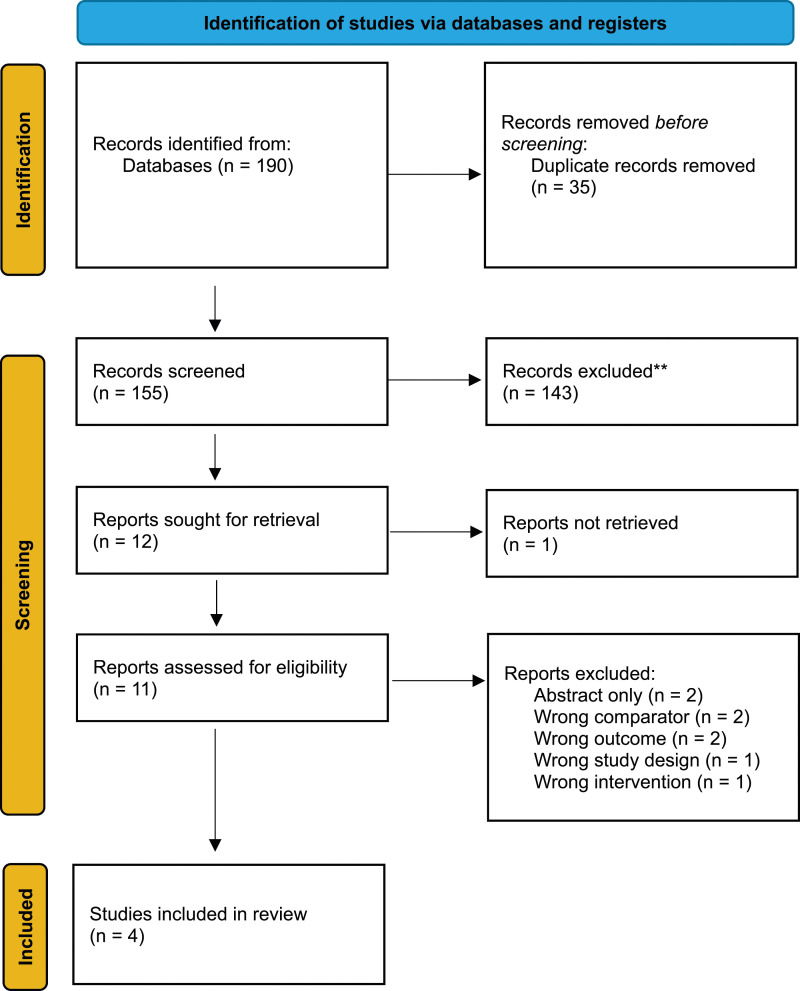
PRISMA flow diagram demonstrating the total number of papers included.

A total of 190 studies were identified in the initial search. We excluded 186 studies after abstract and full-text screening. Four studies, comprising 363 patients, were therefore included in this review (Table 1).^10–13^ A narrative synthesis was conducted, given the results were not reported in a format suitable for formal meta-analysis (Figure 1).

**Table 1.** Summary of papers included and their demographic. *M = male, F = female, DUS = duplex ultrasound*.

Author	Year	Country	Study design	Study type	Participants	Gender (M:F)	Age	Comparator
Kajeweska et al	2023	Poland	Multicentre	Prospective	61	14:47	-	DUS
Soffer et al	2021	USA	Single centre	Retrospective	101	14:87	51	DUS
Soffer et al	2020	USA	Single centre	Retrospective	102	14:87	49	DUS
Davalos et al	2023	Argentina	Single centre	Retrospective	99	50:49	56.6	DUS

### Detection of superficial insufficiency

Two studies found a significant correlation between extent of reflux and mean temperature of the affected area with use of thermal imaging, whereby increased temperature was noted along the vein in areas of SVI previously confirmed on a duplex scan (*p* < .05).^10,11^ There was a positive correlation between increasing mean temperature and increased CEAP classification recorded in a small group of patients noted in one study, although the sample size was noted to act as a limiting factor when reporting this finding (*p* < .05).^10,11^

One study identified an average increase in the total detection of venous reflux by introducing thermal imaging to the routine clinical assessment of duplex ultrasound of 24.6%, when compared to previous scans for their cohort of patients (*p* = .015, 95% CI 6.4, 61).^12^ Thus, introducing thermal imaging as a precursor to duplex was associated with a significant increase in clinically relevant incompetent segment detection. This study identified a difference in detection based on anatomical location of venous insufficiency. The frequency of detection of anterior accessory great saphenous vein doubled from 10 to 20 and posterior accessory great saphenous vein tripled from 2 to 6. However, no statistical analysis was conducted as the numbers were too small to be significant. This same study also noted a statistically significant increase in the detection of small saphenous vein incompetence of 36% that was found on a repeat DUS, but not the initial scan, when using thermal imaging to the clinical assessment pathway (*p* = .030) (95% CI 1.08, 5.12).

### Sensitivity and specificity

One study reported the sensitivity and specificity of thermal imaging in comparison to use of duplex.^13^ The sensitivity and specificity of thermal imaging to detect superficial insufficiency were 98.30% (95% CI, 95.2%–99.4%) and 100% (95% CI 85.7%–100%), respectively. Overall accuracy was noted as 98.5% (95% CI, 95.7%–99.7%).

### Risk of bias

Four non-randomised studies were included in this systematic review and were assessed using the ROBINS-I tool. The overall risk of bias was moderate in three of four studies. One study was found to have a serious overall risk of bias because of the risk of selection bias and missing data. Details of individual components of the assessment are provided (Supplemental Data, Figure 1).

## Discussion

This is the first systematic review evaluating the use of thermal imaging in superficial venous disease. The renewed popularity in thermal imaging has allowed for newer, more robust evidence that suggests the applicability and usefulness of thermal imaging as a diagnostic device in multiple pathologies, albeit not specific to SVI. The evidence at present shows promising application of thermal imaging in venous disease that could be used in clinical practice. However, the similar patterns between thermograms and duplex scans provides data to support further prospective studies to determine the role of thermal imaging in the decision-making process for whether patients need a formal duplex scan or a referral for vascular services.

Given the limited evidence in thermal imaging, there is currently no stated reporting outcomes, attributing the varied results reported in this systematic review and warranting further investigation. Similarly, a lack of characteristic reporting across all papers that is typically required for SVI diagnoses was noted. At present, there appear to be no evidence-based recommendations on how to present thermography data for quantitative analysis of SVI disease. The lack of uniform reporting for areas of reflux, including whether the DUS and thermal imaging correlated to GSV or SSV reflux, was noted in the majority of papers. Due to this, it was difficult to ascertain where the SVI was present within the cohort groups, subsequently making it harder to pool data and reach a meaningful clinical conclusion. This could be addressed in future work assessing different CEAP classifications to the thermography changes noted, to establish if detectable differences are present and thereby attempt to form an evidence-based reporting outcome. A large prospective study could provide the additional evidence needed to further validate the use of thermal imaging as a screening tool. The inclusion of a primary outcome of the negative likelihood ratio (NLR) would be most beneficial when reviewing a screening tool, alongside further assessment of the sensitivity and specificity. Given the prospective nature of the proposed study, accurate reporting of the range of outcomes noted in all previous reviews could then allow for subsequent analysis to determine those that are most accurate in detecting SVI with thermal imaging.

This review has several strengths. The comprehensive and systematic approach for identifying the included studies makes it unlikely that relevant studies were missed. All steps in our methodology, including screening, study selection, data extraction and risk of bias were performed independently and in duplication to minimise any potential bias and reduce subjectivity. We also assessed the certainty of the included articles to identify all sources of bias using the relevant tool. This is the first review to evaluate the current literature and explore the outcomes of utilising thermography as a diagnostic tool, with reference to sensitivity and specificity in comparison to the gold-standard, duplex ultrasound. However, we are aware that a key limitation in this review is the inclusion of non-randomised cohort studies with small sample sizes, which inherently carry a potential risk of bias. Also, we were unable to conduct a meta-analysis/pooled diagnostic accuracy analysis because outcomes were only reported in one study and the outcome measures reported in the other studies were not sufficiently similar to be combinable. It is worth noting that the retrospective nature of the majority of papers included in this review highlights a lack of evidence towards the applicability of thermal imaging as a screening tool. However, we have noted this in the risk of bias assessment.

## Conclusion

Thermal imaging has the ability to produce heat maps that may mirror areas of venous reflux, thus suggesting the possibility of its use as a screening tool in the management of patients. This review has highlighted the need to carry out further large-scale prospective studies to assess the diagnostic accuracy of advanced thermal imaging technologies as compared to DUS. Given the increasing pressures within healthcare systems to deliver patient care and diagnostic tests, the availability of a fast, cheap and highly specific rule out test such as might be possible with thermography for SVI would be incredibly useful. Currently, only four small-scale studies exist within the literature and only one diagnostic accuracy studies, leaving much to be desired in terms of technological validation. Our review has highlighted that there is no existing research comparing thermograms between different venous pathologies and, thus, emphasises the need for such work to be carried out. This highlights a promising avenue of research that could lead to the development of an integrated screening tool, ultimately helping to optimise the diagnostic pathway for patients with SVI.

## Supplemental Material

Supplemental Material - Thermal imaging as a diagnostic tool for superficial venous insufficiency – a systematic review Supplemental Material for Thermal imaging as a diagnostic tool for superficial venous insufficiency – a systematic review by Marwah Salih, Sarah Salih, Benedict R H Turner, Sarah Onida, and Alun H Davies in Phlebology

## References

[1] Davies AH . The seriousness of chronic venous disease: a review of real-world evidence. Adv Ther 2019; 36(Suppl 1): 5–12. 30758738 10.1007/s12325-019-0881-7 PMC6824448

[2] Salim S Machin M Patterson BO , et al. Global epidemiology of chronic venous disease: a systematic review with pooled prevalence analysis. Ann Surg 2021; 274(6): 971–976. 33214466 10.1097/SLA.0000000000004631

[3] Barnes GD Gafoor S Wakefield T , et al. National trends in venous disease. J Vasc Surg 2010; 51(6): 1467–1473. 20304580 10.1016/j.jvs.2009.12.070

[4] Eklof B Rutherford RB Bergan JJ , et al. Revision of the CEAP classification for chronic venous disorders: consensus statement. J Vasc Surg 2004; 40(6): 1248–1252. 15622385 10.1016/j.jvs.2004.09.027

[5] Khilnani NM Min RJ . Imaging of venous insufficiency. Semin Intervent Radiol 2005; 22(3): 178–184. 21326691 10.1055/s-2005-921950 PMC3036278

[6] Robertson L Lee AJ Evans CJ , et al. Incidence of chronic venous disease in the Edinburgh Vein Study. J Vasc Surg Venous Lymphat Disord 2013; 1(1): 59–67. 26993896 10.1016/j.jvsv.2012.05.006

[7] Ring EFJ Ammer K . The technique of infrared imaging in medicine*. In: Infrared Imaging [Internet]. Bristol: IOP Publishing, 2015. Available from. DOI: 10.1088/978-0-7503-1143-4ch1.

[8] Ring EF . The historical development of thermometry and thermal imaging in medicine. J Med Eng Technol 2006; 30(4): 192–198. 16864230 10.1080/03091900600711332

[9] Tattersall GJ . Infrared thermography: a non-invasive window into thermal physiology. Comp Biochem Physiol Mol Integr Physiol 2016; 202: 78–98. 10.1016/j.cbpa.2016.02.022 26945597

[10] Davalos MPA Brioschi ML da Rosa SE , et al. Can dual infrared-visual thermography provide a more reliable diagnosis of perforator veins and reflux severity? J Clin Med 2023; 12(22): 7085. 38002697 10.3390/jcm12227085 PMC10672064

[11] Kajewska J Stanek A Sieroń K , et al. May thermal imaging be useful in early diagnosis of lower extremities chronic venous disease? Pol J Med Phys Eng 2023; 29(1): 73–84.

[12] Soffer A . Thermal imaging of superficial leg circulation improves venous diagnostic efficiency and completeness. Vascular Disease Management 2020; 17: E208–E211.

[13] Soffer A . Sensitivity and specificity of thermal imaging when used to detect superficial venous reflux as compared to duplex ultrasound. Vascular Disease Management 2021; 18: E45–E49.

