# Supplementary material for: Thermal imaging as a diagnostic tool for superficial venous insufficiency – a systematic review
Source: Phlebology. 2024 Nov 12;40(4):223–7. doi: 10.1177/02683555241301194 (PMC11994872; doi:10.1177/02683555241301194)
Supplement: Supplemental Material - Thermal imaging as a diagnostic tool for superficial venous insufficiency – a systematic review [file sj-pdf-1-phl-10.1177_02683555241301194.pdf]

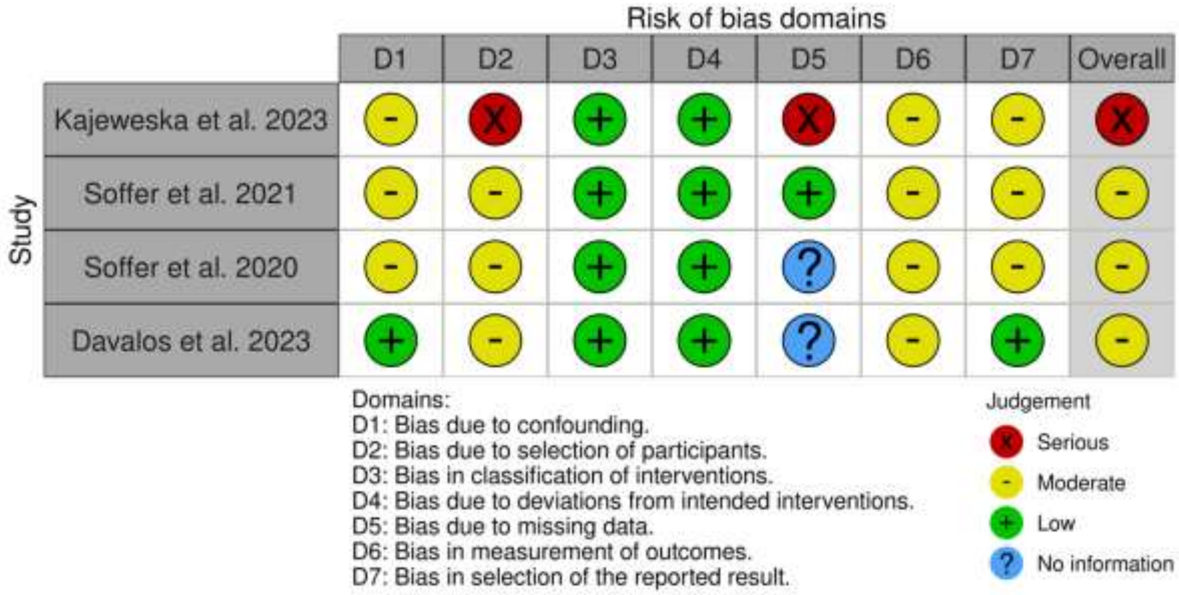

S Figure 1: ROBIN-I tool for assessment of papers included in review**.**
